# Comparative pathogenicity of very virulent and novel variant infectious bursal disease virus Egyptian strains in chickens

**DOI:** 10.1186/s12985-026-03111-7

**Published:** 2026-03-22

**Authors:** Mahmoud Ibrahim, Dalia Ayman, Walaa Arafa, Marwa A. Abdelmagid, Ahmed A. El-sanousi, Mohamed Shaheen

**Affiliations:** 1 Department of Birds and Rabbit Medicine, Faculty of Veterinary Medicine, University of Sadat City, Sadat, 32958 Menoufia Egypt; 2 Egyptian Company for Biological & Pharmaceutical Industries (Vaccine Valley), 6th of October City, Giza, 12511 Egypt; 3 Reference Laboratory for Veterinary Quality Control on Poultry Production, Agricultural Research Center, Animal Health Research Institute, Dokki, Giza, 12618 Egypt; 4 Department of virology, Faculty of veterinary Medicine, Cairo University, Giza, Egypt; 5 Environmental Virology laboratory, Environment and Climate Change Research Institute, National Research Centre, Dokki, Giza, 12622 Egypt

**Keywords:** VvIBDV, nVarIBDV, Pathogenicity, Bursa, Chickens, Egypt

## Abstract

Infectious bursal disease (IBD) is an important viral disease that causing severe immunosuppression in young chickens. This current study compared the pathogenicity of very virulent IBDV (vvIBDV) and Chinese novel variant IBDV (nVarIBDV) strains in Specific Pathogen-Free (SPF) chickens. Chickens infected with the nVarIBDV strain exhibited no clinical signs or mortality, with mild edema and swelling in bursas during early infection, followed by progressive atrophy by the seventh- and fifteenth-days post-infection (dpi). In contrast, vvIBDV displayed severe systemic disease, with early onset ruffled feathers, hemorrhages on the thigh muscles. While both strains caused bursal atrophy, the vvIBDV strain induced more severe systemic pathology, characterized by hemorrhages in bursa, renal and hepatic degeneration, with 50% mortality. The vvIBDV induced severe reduction in Bursa-to-Body Weight (B/BW) ratio, reaching 1.042 ± 0.302, indicating significant immunosuppressive effect. While, the nVarIBDV-infected group showed marked reduction in the B/BW ratio to 1.269 ± 0.269, showing 70% decrease compared to the control group. The results of histopathology showed a mild bursitis, lymphoid depletion, and interfollicular connective tissue proliferation in the nVarIBDV-infected chickens. While, vvIBDV-infected chickens induced a severe bursitis with necrosis of lymphoid follicles and interfollicular fibrous tissue proliferation indicating lymphoid depletion. Moreover, the challenge with nVarIBDV and vvIBDV alter the immune response of a trivalent inactivated vaccine either before vaccination or after vaccination. This study provides valuable data about the pathogenicity of two cocirculating IBDVs in Egypt and highlighted the need for strict routine monitoring for nVarIBDV infection in chicken flocks as it has no clinical signs. Further research may be required to assess the efficacy of the currently available IBD vaccines in Egypt against these strains.

## Introduction

IBDV is a highly contagious immunosuppressive pathogen affecting chickens, leading to significant economic losses in the global poultry industry. It primarily targets actively proliferating B lymphocytes in the Bursa of Fabricius, causing lymphoid depletion, immunosuppression, and increased susceptibility to secondary infections. The disease, commonly known as Gumboro disease, predominantly affects young chickens between three to six weeks of age, a critical period for immune system development. The resulting immunosuppression leads to increased mortality rates, poor vaccine responses, and heightened vulnerability to opportunistic infections, including fowl Adenovirus (FAdV), Newcastle disease virus (NDV), avian influenza virus (AIV), and bacterial pathogens such as *Escherichia coli* and *Mycoplasma gallisepticum* [4, 19]. So, more studies needed on IBDV for understanding its evolution, pathogenic mechanisms, and effective strategies control.

IBDV is a member of the *Birnaviridae* family, classified under the genus *Avibirnavirus*. It possesses a bi-segmented double-stranded RNA (dsRNA) genome enclosed within a non-enveloped icosahedral capsid. Segment A encodes the major structural and non-structural proteins, including VP2, VP3, VP4, and VP5, while segment B encodes VP1, an RNA-dependent RNA polymerase (RdRp) essential for viral replication (Mahgoub, 2012). Among these proteins, VP2 is the primary determinant of antigenicity and immunogenicity. The hypervariable region (HVR) of VP2 undergoes frequent mutations, leading to antigenic drift and the emergence of escape mutants that complicate vaccine efficacy [18]. The RdRp, encoded by segment B, lacks proofreading activity, contributing to a high mutation rate that facilitates viral evolution, reassortment, and adaptation to different host immune pressures [29].

There are two recognized serotypes of IBDV: serotype I, which is pathogenic to chickens, and serotype II, which has been isolated from turkeys but does not cause disease in chickens [17]. Within serotype I, IBDV strains are categorized into different pathotypes based on their virulence and antigenic properties. Classical IBDV (cIBDV) strains were initially controlled using conventional vaccines, but antigenic drift has led to the emergence of variant IBDV strains capable of evading classical vaccine-induced immunity (Jackwood & Sommer-Wagner, 2011). The very virulent IBDV (vvIBDV) strains emerged in Europe during the late 1980s and have since spread globally, causing high mortality rates of up to 60% in susceptible flocks [27]. More recently, novel variant IBDV (nVarIBDV) strains have been emerged in China [30]. The nVarIBDV exhibiting distinct antigenic properties with tendency to evade existing vaccines, complicating disease control efforts.

The global circulation of the nVarIBDV with its immunosuppressive effect make it one of the most important recently emerging viral disease to poultry production worldwide. During 2022, the nVarIBDV was detected and characterized in Argentina and Kazakhstan ([11]; Zikibayeva et al., [36]). In Egypt, first detection of the nVarIBDV of the genogroup A2dB1b was reported during February 2023 [13]. Interestingly, circulating of different IBDV pathotypes include classical virulent, attenuated, very virulent, and novel variant strains, reflecting the dynamic nature of viral evolution in the Middle East region [22]. Coinfection between two IBDV strains from different sub-groups of IBDVs was reported in South Korea [26], that could enhance the virus evolution.

Pathogenicity of IBDV is primarily associated with its ability to induce severe lymphoid depletion in the Bursa of Fabricius, leading to immunosuppression and increased mortality. The severity of bursal atrophy and immune suppression depends on the virulence of the strain. vvIBDV strains are known for their aggressive pathology, characterized by extensive hemorrhages, severe bursal necrosis, and multi-organ involvement, leading to high mortality. In contrast, nVarIBDV strains often cause subclinical or mild disease but with profound immunosuppressive effects, compromising the long-term immune function of infected birds [3, 12, 14, 25]. While vvIBDV induces rapid and severe bursal lesions, nVarIBDV is associated with delayed bursal atrophy, making birds more susceptible to secondary infections and vaccine failures.

Despite widespread vaccination efforts, IBDV remains a persistent challenge due to its high mutation rate, antigenic variation, and immune evasion strategies. Live-attenuated, inactivated, and recombinant vaccines have been widely used for control; however, the continuous emergence of antigenic variants has limited their long-term effectiveness. Mutations in the VP2 protein contribute to immune escape, and the lack of cross-protection among different IBDV strains complicates vaccine development. Current vaccines based on classical strains may not provide adequate protection against emerging vvIBDV and nVarIBDV strains, leading to recurrent outbreaks even in vaccinated flocks [7, 18]. The co-circulation of multiple IBDV strains within poultry populations further complicates disease control, highlighting the need for continuous epidemiological monitoring and improved vaccine formulations.

Given the increasing prevalence of vvIBDV and nVarIBDV strains in Egypt, it is crucial to assess their pathogenicity to understand their impact on poultry health and vaccine efficacy. So, the objective of this study was to compare the pathogenicity of these two circulating IBDV strains in SPF chickens, focusing on clinical manifestations, bursal lesions, and immunosuppressive effects. Understanding the comparative pathogenicity of these strains will aid in the development of effective vaccines and disease management programs to control IBDV outbreaks. Moreover, another experiment to assess the effect of challenge with either IBDV strains on the immune response of inactivated vaccines before or after vaccination.

## Materials and methods

### Viruses

Two distinct strains of IBDV: vvIBDV strain (Accession No. PP785758) and nVarIBDV strain (Accession No. OR594275.1) were used for challenge of chickens. The vvIBDV strain was isolated from infected backyard layer flock that not vaccinated with IBDV live vaccines in 2023 from Menoufia governorate, while, the nVarIBDV strain was isolated from broiler farm vaccinated with Vaxxitek in 2023 from Giza governorate (Abd El Fatah et al., 2024). These strains were isolated, molecularly characterized, and propagated at Vaccine Valley Laboratories (Giza, Egypt). The virus strains were propagated in 9–11-day-old SPF embryonated chicken eggs via the chorioallantoic membrane route (CAM), following standard protocols ([21]; OIE, [33]). The embryos were monitored for characteristic IBDV lesions, including stunted growth, hemorrhages, and death within 48–72 h post-inoculation. Virus stocks of the 3rd embryo passage were titrated using the 50% Embryo Infective Dose (EID₅₀) method, according to Reed and Muench [20]. The viral titers obtained for both strains were more than 10⁵ EID₅₀/ml, and each chick in the experimental infection groups received an oral inoculum of 10³ EID₅₀ in 0.5 ml.

### Experimental birds

A total of 180 SPF White Leghorn chicks were obtained from the Kom-Osheim SPF farm (Fayoum, Egypt). SPF chicks were used to eliminate potential maternal antibody interference, ensuring an accurate assessment of viral pathogenicity [32]. Chicks were housed in a biosafety level-2 (BSL-2) facility and maintained under controlled environmental conditions with drinking water and feed were supplied ad libitum. All chicks were observed for 7 days before the experiment to ensure their health status, and no signs of infection or immunosuppression were observed.

### First experiment

Hundred SPF chicks (21 days old) were randomly divided into three groups; group 1: Chicks were orally inoculated with 10³ EID₅₀ of the nVarIBDV strain (*n* = 35), group 2: chicks were orally inoculated with 10³ EID₅₀ of the vvIBDV strain (*n* = 35), group 3: Chicks were sham-inoculated with sterile PBS as control (*n* = 30). Chicks were monitored daily for 15 days for clinical signs, morbidity, and mortality. The clinical signs were monitored and scored based on a standardized scoring system as follows: 0, No clinical signs; 1: mild ruffled feathers, slight depression; 2: moderate depression, reduced appetite, diarrhea; 3: severe depression, dehydration, hemorrhagic diarrhea; 4: death. Dead birds were immediately necropsied to assess gross pathological lesions.

#### Bursa-to-body weight index (BBIX) calculation

To assess the impact of infection on the Bursa of Fabricius, five birds from each group were euthanized on day 7 post-infection for bursal weight measurement. The bursa and body weights of all the chickens were determined then Bursa: body weight ratio (B: BW ratios) were calculated according to the following formula: B:BW ratio 5 [bursa weight (g)/body weight (g)] x 1,000.

Also, Bursa-to-Body Weight Index (BBIX) was calculated by dividing BBW for the infected group on the BBW for the control group; [BBIX= (bursa: body weight ratios)/(bursa: body weight ratios in the negative group)], according to [23]. A BBIX value below 0.70 was considered indicative of bursal atrophy [16]. The mean BBIX values for each group were recorded along with their standard deviation (SD). The remaining birds were observed until 15 dpi to evaluate the progression of disease and recovery patterns.

#### Gross pathology

To examine viral-induced tissue damage, three birds from each group were euthanized at 3, 7, and 10 dpi, and tissues (Bursa Fabricius, Spleen, Liver, and Kidney) were examined for gross lesions and part from each organ were collected in 10% buffered formalin for histopathology. Another part from bursa and spleen was collected and preserved at -80 °C to be tested for presence of IBDV RNA by RT-PCR.Dead birds in group 2 challenged by vvIBDV used for gross lesions examination and samples collection. At 15 dpi 3 birds were necropsied and examined grossly.

#### Molecular detection and virus titration

Molecular detection of IBDV virus in bursa and spleen was performed by RT-PCR as previously described [2]. Briefly, RNA was extracted from the bursa and spleen tissue suspensions that used in amplification of 789 bp fragment of IBDV VP2 gene using 2 primers previously designed in-house by RT-PCR. Moreover, virus titration in bursa and spleen tissue suspensions collected at 3rd dpi was performed by inoculating ten-fold serial dilutions (10^− 1^ – 10^− 6^) in 9–11-day-old SPF-ECEs via CAM route using the 50% Embryo Infective Dose (EID₅₀) method, according to Reed and Muench [20].

#### Histopathology

For microscopic analysis, tissues were fixed in 10% neutral-buffered formalin for 24 h then dehydrated in graded ethanol concentrations followed by Embedding in paraffin wax and sectioned into 4–5 μm slices using a microtome and finally stained with hematoxylin and eosin (H&E) for microscopic examination. Histopathological scoring was performed based on lymphoid depletion, necrosis, and inflammatory infiltration, using a standardized grading system [35]. Scoring System (0–4 scale): 0 = Normal (no lesions), 1 = Minimal (< 25% affected), 2 = Mild (25–50% affected), 3 = Moderate (50–75% affected), and 4 = Severe (> 75% affected).

### Second experiment

To assess the effect of both IBDV strains on the immune response of inactivated vaccines, a trivalent vaccine containing inactivated H5N8, H9N2 and Lasota antigens was prepared. Hatchery vaccination for chickens with killed vaccines became more preferable in almost all Middle East countries like Egypt in addition to booster vaccinations in long lived chickens, so this experiment assessed the infection effect of both IBDV strains on the immune response of killed vaccines after and before vaccination. For this experiment, 80-SPF chicks were used and divided into 6 groups as shown in Table 1. Blood samples collected and serum separated to be tested by hemagglutination inhibition assay (HI) according to OIE manual.

**Table 1:** Experimental design for the second experiment:

Group ID	Chicks number**	Age at vaccination (days-old)	Challenge*	Serum collection post vaccination (WPV)
G1	10	7	VarIBDV	3rd, 4th, 5th WPV
G2	10	28		1st, 2nd, 3rd WPV
G3	20	7	vvIBDV	3rd, 4th, 5th WPV
G4	20	28		1st, 2nd, 3rd WPV
G5	10	7	Non-challenged	3rd, 4th, 5th WPV
G6	10	28		1st, 2nd, 3rd WPV

* Challenge was performed at 21-days-old for G1-G4

** G3 and G4 challenged with vvIBDV have 20 chicks as this virus cause about 50% mortalities. WPV: weeks post vaccination

### Statistical analysis

All analyses were conducted using R version 4.5.1. Serological data (HI titers) were analyzed by one-way ANOVA with Tukey’s HSD post-hoc test. Non-parametric histopathological scores were compared using the Kruskal-Wallis test (with Dunn’s post-hoc test) and the Friedman test for time-course analysis. Effect sizes (Cohen’s d) and a post-hoc power analysis was also performed. Significance was set at *p* < 0.05.

## Results

### Clinical signs and post-mortem (PM) lesions

In the nVarIBDV-infected group, there is no clear clinical signs were observed and chicks were normal like birds in the control birds. **By the third dpi**, the Bursa of Fabricius became swollen, with a gelatinous yellowish exudate visible on the serosal surface, but no hemorrhagic lesions were observed. The spleen appeared moderately enlarged and congested, while the liver and kidney showed mild congestion. **By the 7th and 10th dpi**, the bursa became atrophied with adhesion and hardly detached out and the spleen, liver and kidney showed no marked lesions. By the fifteenth dpi, the bursa had undergone severe atrophy, appearing shrunken and fibrotic, indicative of lymphoid depletion and immune suppression. The intestine showed lesions indicating enteritis from 3rd dpi to 15th dpi. No mortality was recorded in this group.

**Fig. 1:**
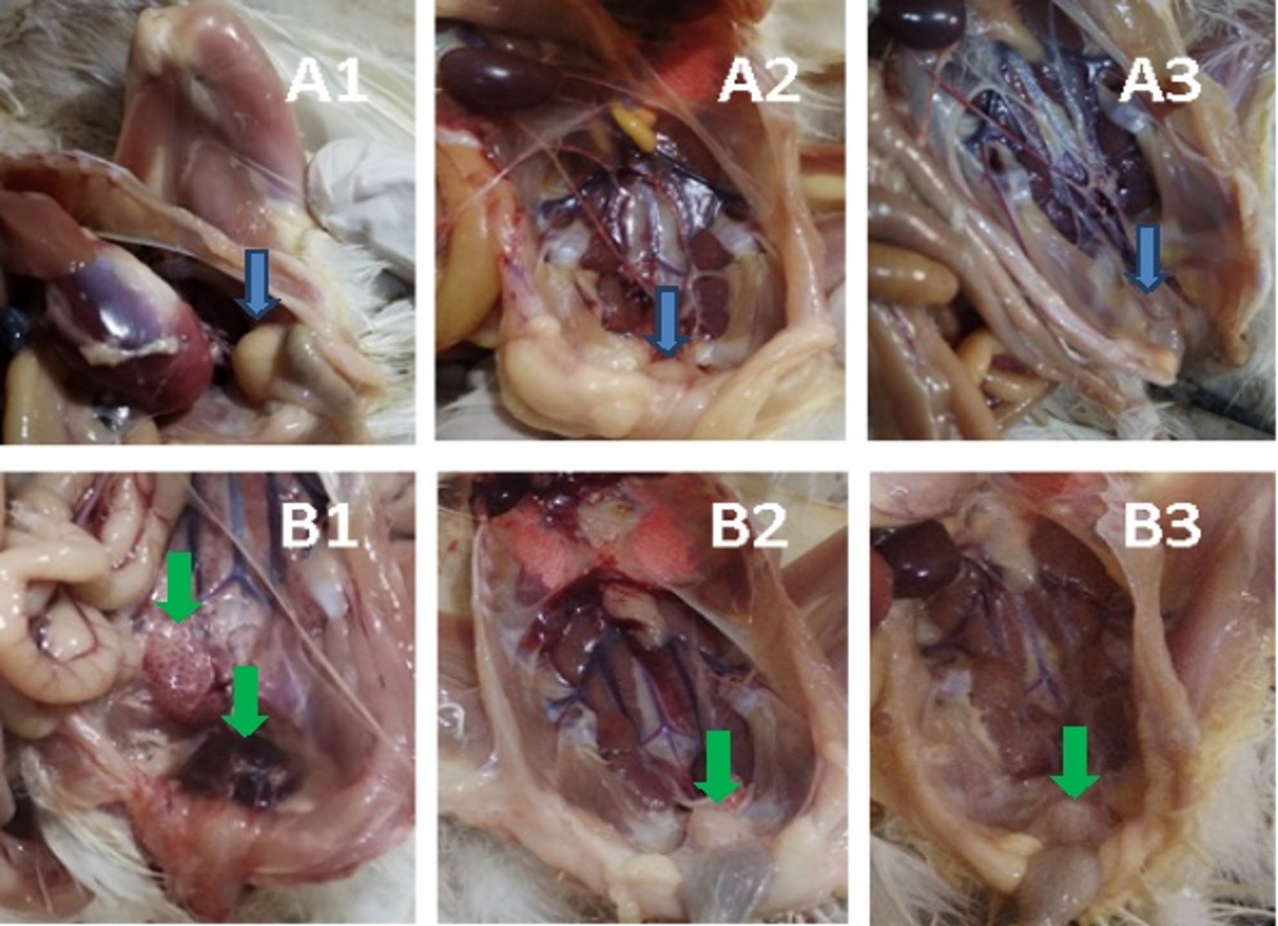
Gross pathological lesions in the nVarIBDV- and vvIBDV-infected groups at different time points post infection. nVarIBDV-infected group: **A1**, **A2**, and **A3**: PM lesions at 3-, 7-, and 10-dpi (Blue arrows indicate bursal atrophy). vvIBDV group: **B1**, **B2**, and **B3**: PM lesions at 3-, 7-, and 10-dpi (Green arrows indicate bursa and kidney gross changes)

In contrast, the vvIBDV-infected group displayed more severe and acute clinical signs, reflecting the increased virulence of the strain. Affected chicks exhibited marked lethargy, progressive weight loss, and severe ruffling of feathers as early as the second dpi. Some birds displayed depression, reluctance to move or laying on one side, and diarrhea with pasty vent staining. **By the second and third dpi**, the bursa appeared hemorrhagic, congested, and distended, resembling a sac filled with blood in some birds or edematous and firm in others. Petechial hemorrhages were observed in the thigh muscles in dead birds. Kidneys were pale and swollen, with prominent urate deposits within the ureters. **By the 7th and 10th dpi**, extensive necrosis and atrophy of the bursa were observed with adhesion to adjacent tissues. The spleen and liver showed congestion and/or necrosis. The kidneys exhibited nephrosis, and appeared pale and swollen. **By the fifteenth dpi**, the bursa had undergone complete fibrotic atrophy, appearing as a shrunken, fibrotic remnant with no visible lymphoid tissue. The spleen, liver and kidney showed no clear PM lesions compared to noninfected birds. The control group exhibited no significant clinical signs or post-mortem lesions. These gross pathological changes are illustrated in Fig. 1.

The vvIBDV group showed rapid onset of severe clinical signs by DPI 3 (mean clinical score 2.34 ± 0.21), while the variant-infected group exhibited only mild signs (mean clinical score 0.71 ± 0.15). Control birds remained normal throughout the observation period.

The vvIBDV group exhibits rapid onset of severe bursal lesions by DPI 3 (mean 2.85 ± 0.18) progressing to complete fibrotic atrophy (mean 3.0) by 15th dpi. The variant strain shows initial bursal swelling at 3 dpi (mean 2.1) followed by progressive atrophy. Control birds show no bursal lesions throughout the study.

### Mortality

**Fig. 2:**
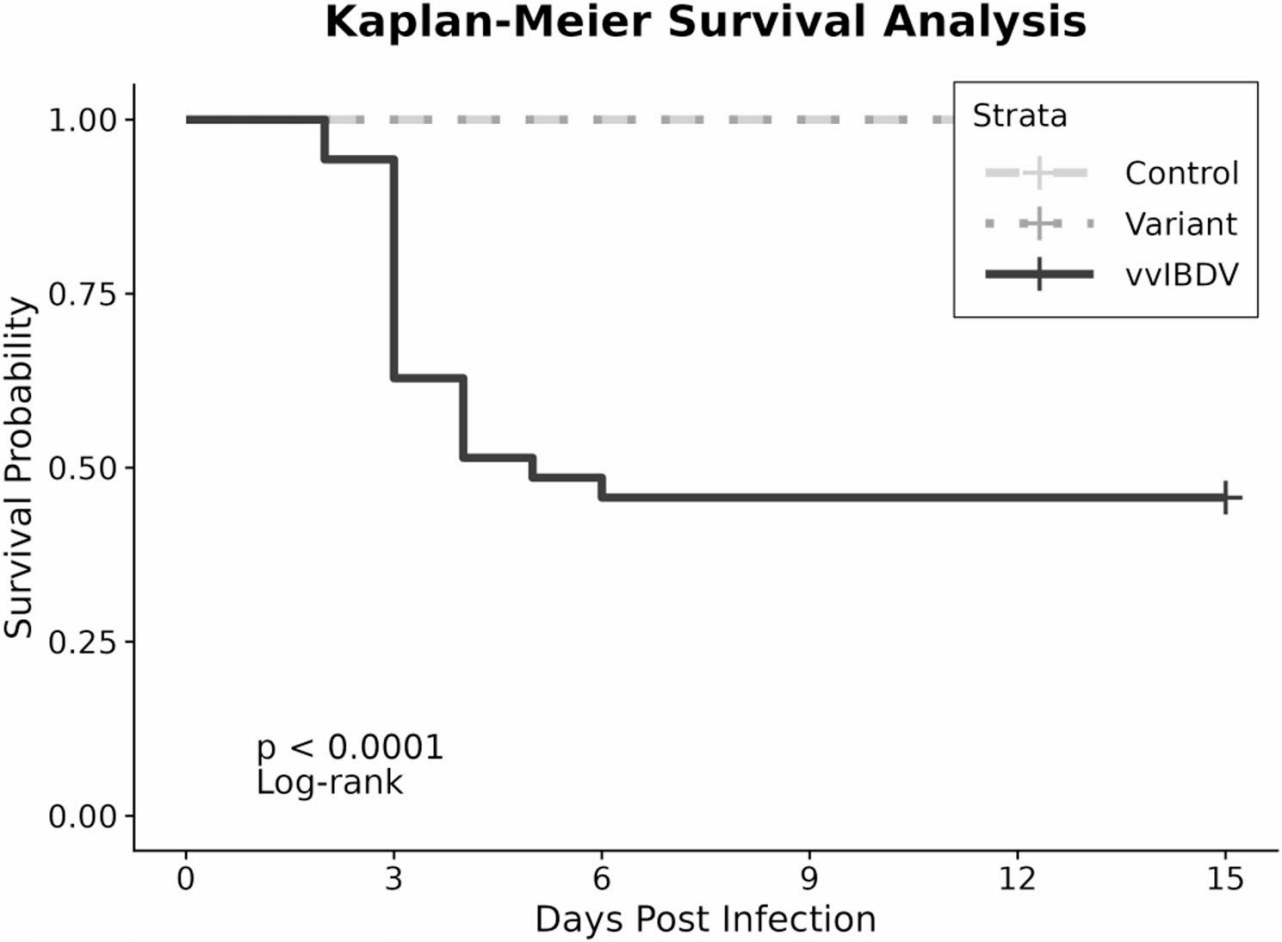
Kaplan-Meier survival analysis of IBDV-infected chickens over 15 days. The vvIBDV group shows rapid mortality with median survival time of 3 days and survival probability dropping to 45.7% by 15th dpi. Log-rank test comparing survival curves shows highly significant differences (χ² = 40.2, *p* < 0.001)

No mortality was recorded in either the control or nVarIBDV-infected groups. However, in the vvIBDV-infected group, mortality reach 54.3% (19/35 chicks), with deaths occurring primarily between the 2nd and the 6th dpi. Two birds died in the 2nd dpi, 11 birds died in the 3rd dpi, 4 birds died in the 4th dpi, 1 bird died in the 5th dpi and 1 bird died in the 6th dpi. The peak mortality was between 2nd and 4th dpi with the highest mortality observed in the 3rd dpi. The survival curves illustrate the distinct virulence patterns: vvIBDV causes acute fatal disease, while the variant strain is non-lethal despite causing pathology (Fig. 2).

### Bursa-to-body weight ratio

**Fig. 3:**
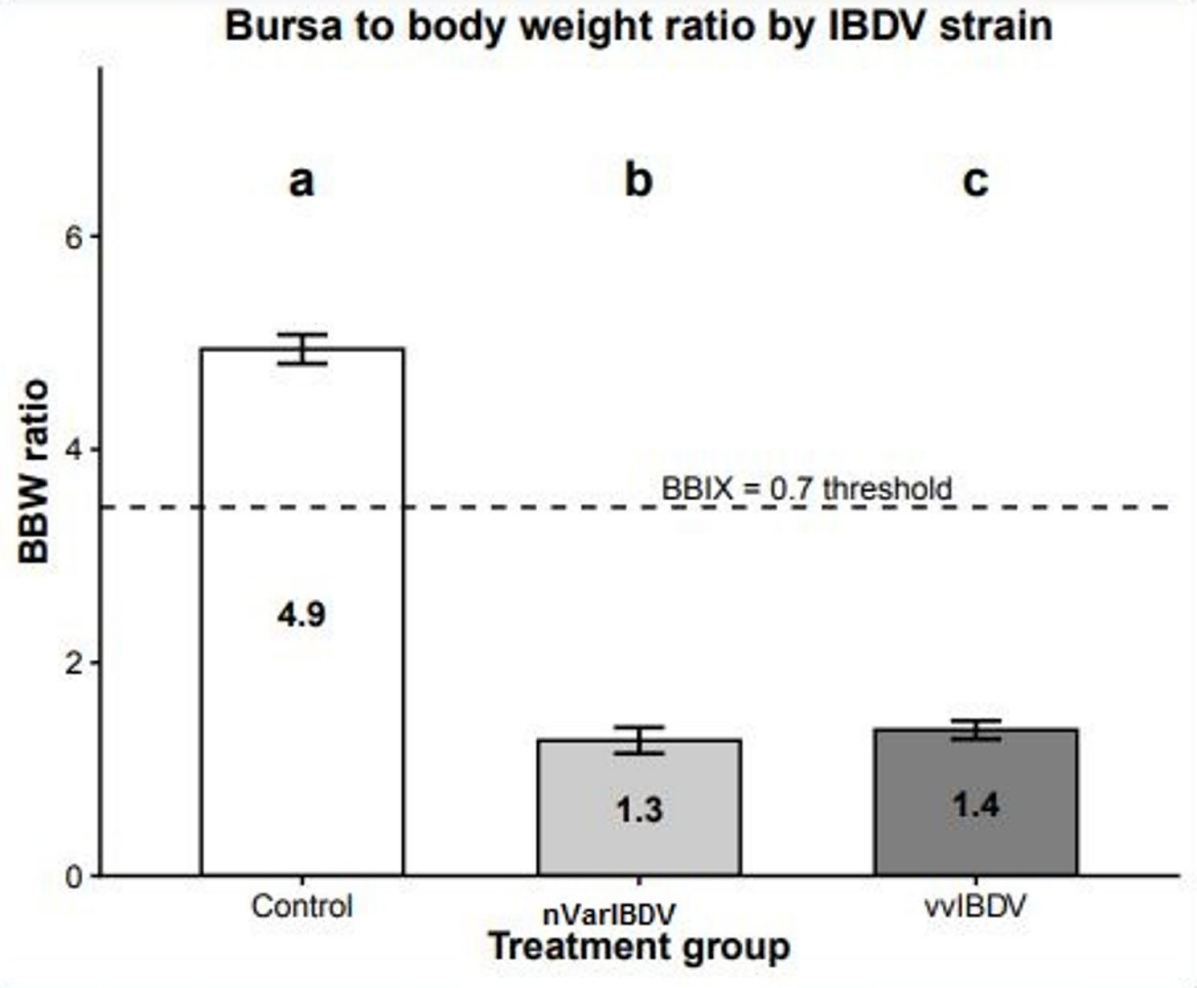
Bursa-to-Body Weight (B/BW) ratio at 7th dpi, illustrating significant bursal atrophy in nVarIBDV^b^ and vvIBDV^c^ infected chickens compared to control noninfected birds

To evaluate the extent of bursal atrophy, autopsies were performed on five chicks per group at seven days PI. The control group exhibited a mean Bursa-to-Body Weight (B/BW) ratio of 4.974 ± 0.2478, which is consistent with normal bursal development. In contrast, the nVarIBDV- and vvIBDV-infected groups demonstrated significant bursal atrophy, with a marked reduction in the B/BW ratio to 1.269 ± 0.269, and 1.369 ± 0.302, respectively, indicating more than a 70% decrease compared to the control group as shown in (Fig. 3). Moreover, the BBIX for the nVarIBDV- and vvIBDV-infected groups was 0.257, and 0.277, respectively, and both were below 0.7. These findings confirm that both the nVarIBDV and vvIBDV strains induce obvious bursal atrophy, though the vvIBDV strain caused more drastic and rapid tissue destruction.

### Molecular detection and titration of IBDV in tissues

The RNA of IBDV was detected in bursa and spleen tissues from birds infected with nVarIBDV and vvIBDV at 3rd, 7th, and 10th dpi. Moreover, viral load in bursa and spleen collected at 3rd dpi measured by calculating the mean embryo infective dose (EID50)/ml tissue suspension; in nVarIBDV-infected chickens (bursa = 4.25 log10 and spleen = 3.15 log10), while in vvIBDV infected chickens (bursa = 6.43 log10 and spleen = 3.85 log10).

### Histopathology

**Fig. 4:**
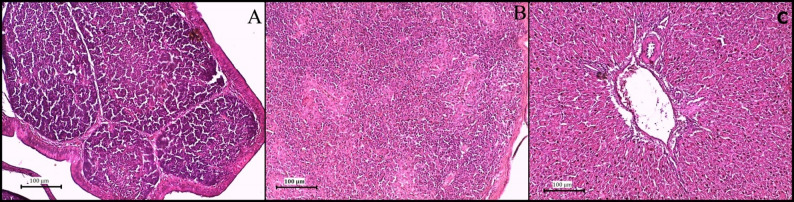
photomicrograph of nonchallenged control group (H&E), bursa (**A**), spleen (**B**), liver (**C**)

Histopathological examination revealed distinct pathological changes in the Bursa of Fabricius, spleen, liver, and kidneys across the experimental groups. Organs from the control group exhibited normal histological structures, with intact lymphoid follicles in the bursa and no signs of inflammatory changes (Fig. 4).

**Fig. 5:**
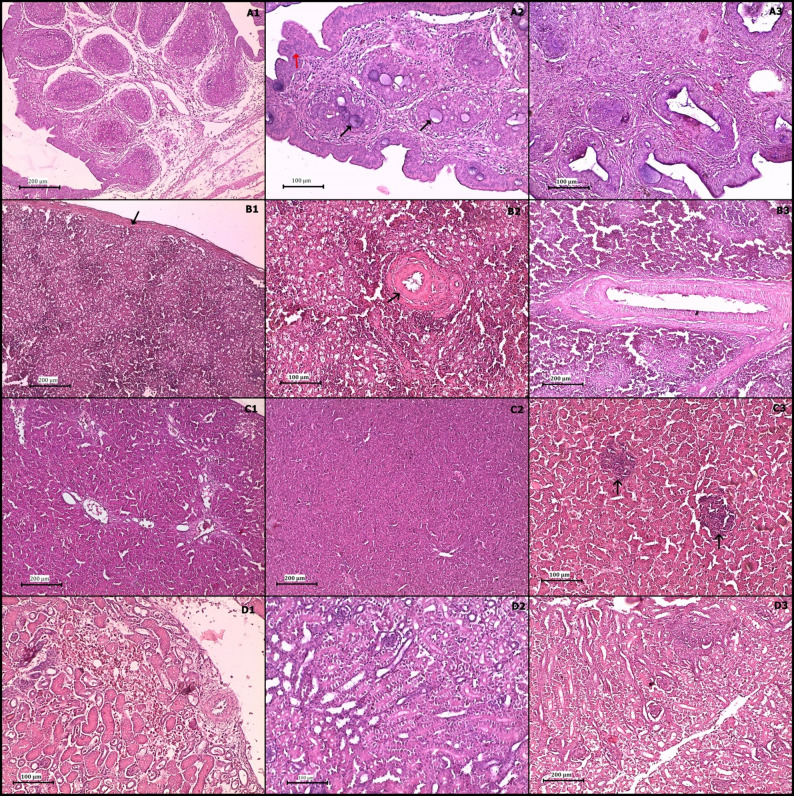
photomicrograph of vvIBDV challenged group (H&E); bursa (**A1**-**A3**), spleen (**B1**-**B3**), liver (**C1**-**C3**), and kidney (**D1**-**D3**) at 3rd, 7th, and 10th days post infection (dpi), respectively

In the vvIBDV-infected group, the bursa displayed severe bursitis with necrosis of lymphoid follicles and interfollicular fibrous tissue proliferation by the third dpi. By the seventh dpi, cortical epithelium proliferation, intrafollicular necrotic cysts, and complete lymphoid depletion were evident. By the tenth dpi, the bursa had undergone massive lymphoid depletion, with replacement by fibrous connective tissue and severe interlobular edema. The spleen exhibited splenitis, with diffuse lymphoid depletion and a mildly thickened capsule by the third dpi. By the tenth dpi, the spleen displayed severe vasculitis, hemorrhages, and lymphoid depletion. The liver showed diffuse hepatocytic degeneration and central vein congestion by the third dpi, progressing to extensive necrosis and perivascular lymphocytic infiltration by the tenth dpi. The kidneys exhibited diffuse nephrosis, intertubular edema, and inflammatory infiltration by the third dpi, progressing to severe tubular degeneration by the tenth dpi, indicating severe renal dysfunction (Fig. 5).

**Fig. 6:**
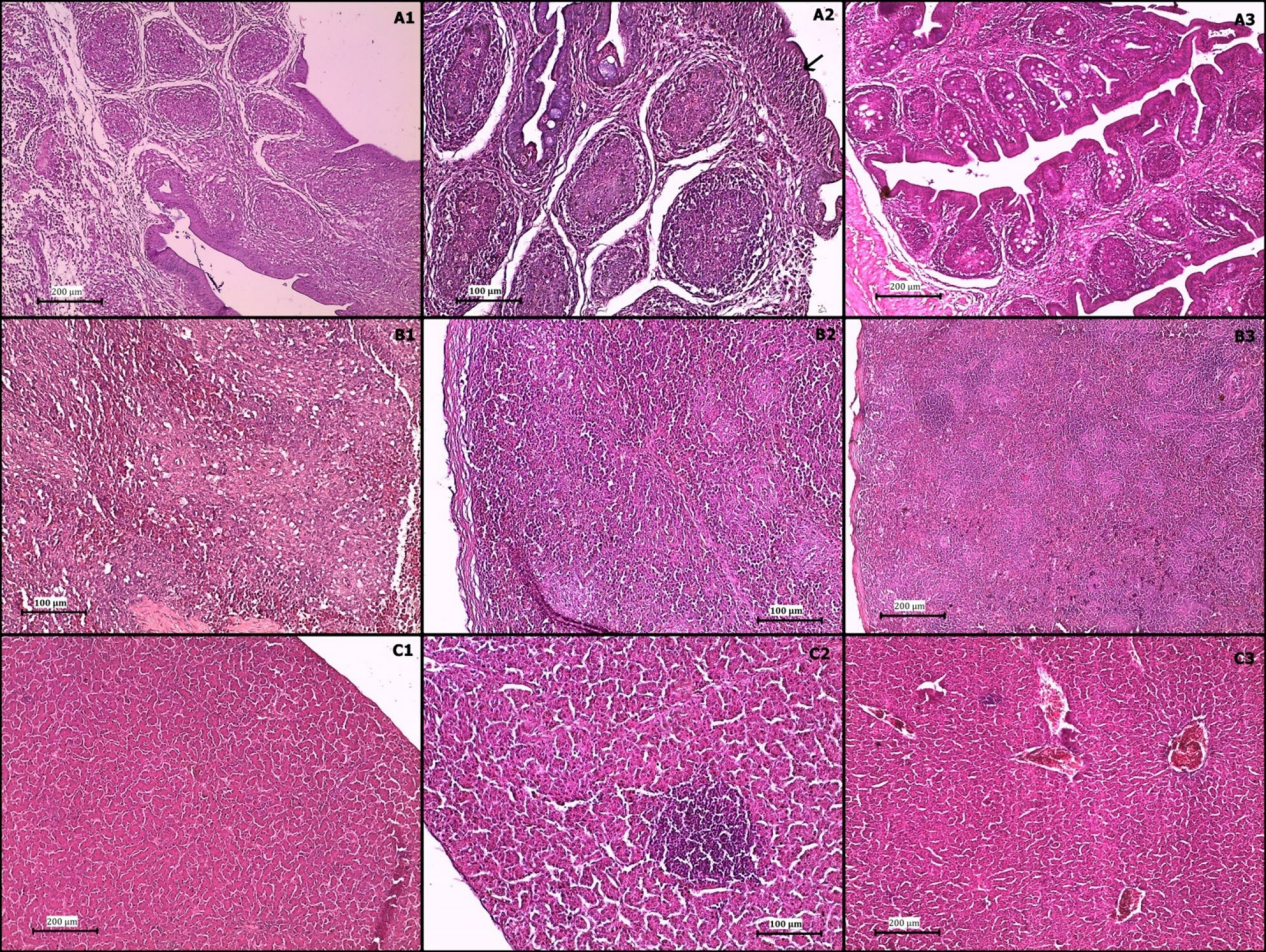
photomicrograph of nVarIBDV challenged group (H&E); bursa (**A1**-**A3**), spleen (**B1**-**B3**), and liver (**C1**-**C3**) at 3rd, 7th, and 10th dpi, respectively

In the nVarIBDV-infected group, the bursa exhibited mild bursitis, lymphoid depletion, and interfollicular connective tissue proliferation by the third dpi. By the seventh dpi, hyperplasia of the cortical epithelium, interfollicular fibrous tissue proliferation, and moderate lymphoid depletion were observed. By the tenth dpi, the bursa showed cystic degeneration, medullary fibrosis, and severe depletion of lymphoid follicles, though the structural integrity of the organ remained partially preserved. A comparative analysis showed that vvIBDV-infected chickens had bursas with severe interlobular edema and complete lymphoid depletion, while nVarIBDV-infected chickens had bursas with retained some epithelial hyperplasia, indicating less extensive destruction (Fig. 6).

**Fig. 7:**
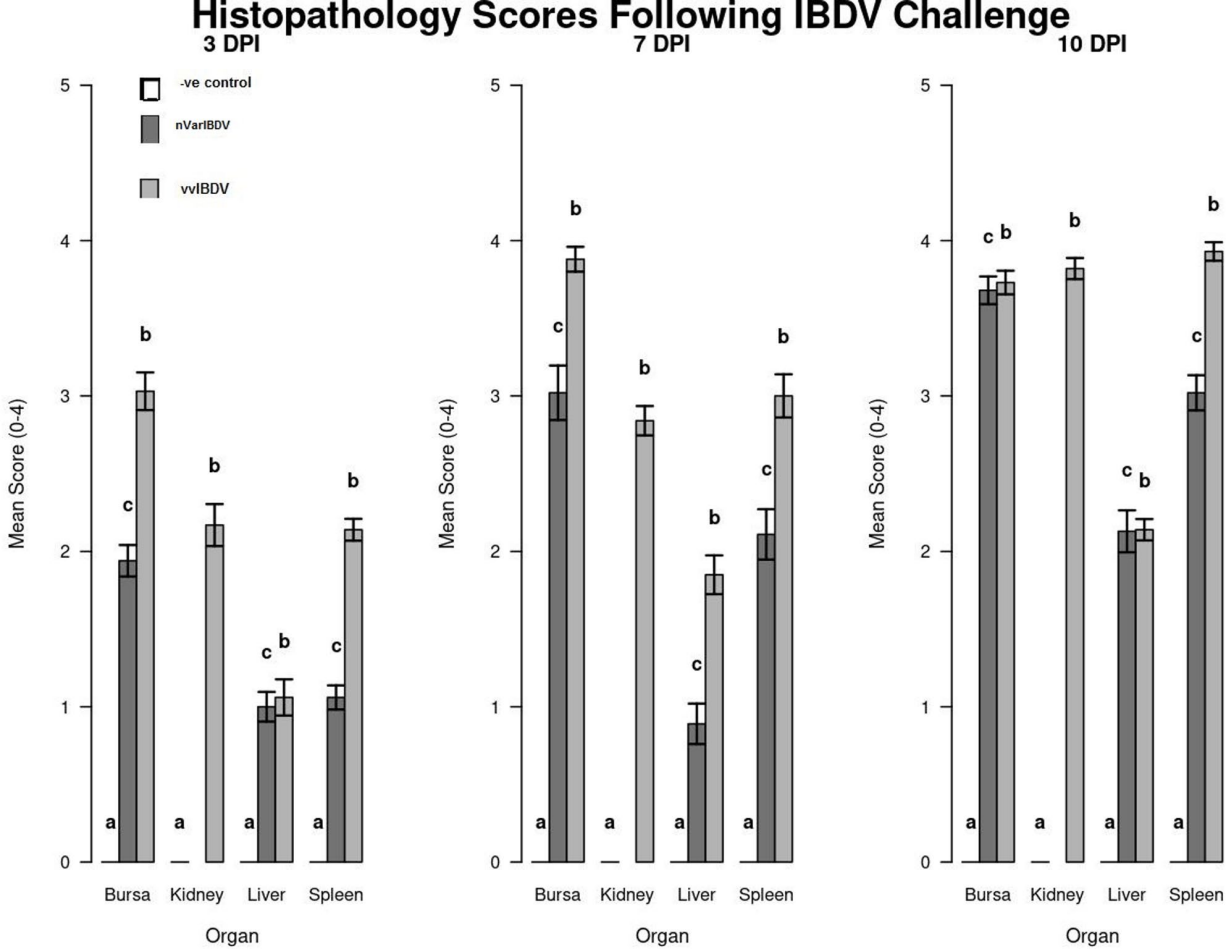
Mean lesion scores for histopathology of different organs in different groups with its statistical analysis

Mean lesion score of the histopathological findings for all tested organs at 3rd, 7th, and 10th dpi was high in vvIBDV infected chickens compared to nVarIBDV group with significan difference (*P* < 0.05). Both IBDV infected groups showing Highly significant difference (*p* < 0.001) compared to control noninfected group (Fig. 7).

### Serological response of inactivated vaccine in IBDV challenged chickens

**Fig. 8:**
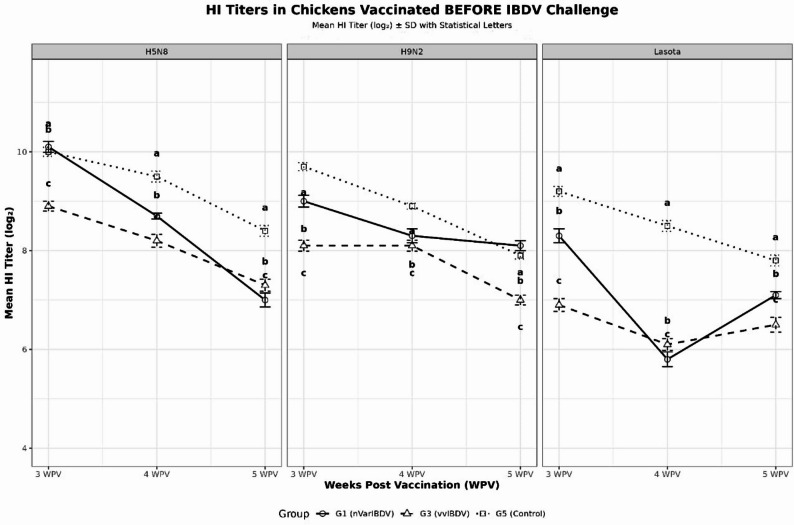
Mean HI titers (log2) for chicken groups vaccinated before challenge. Superscripts (**a**, **b**, **c**) indicate statistical differences

**Fig. 9:**
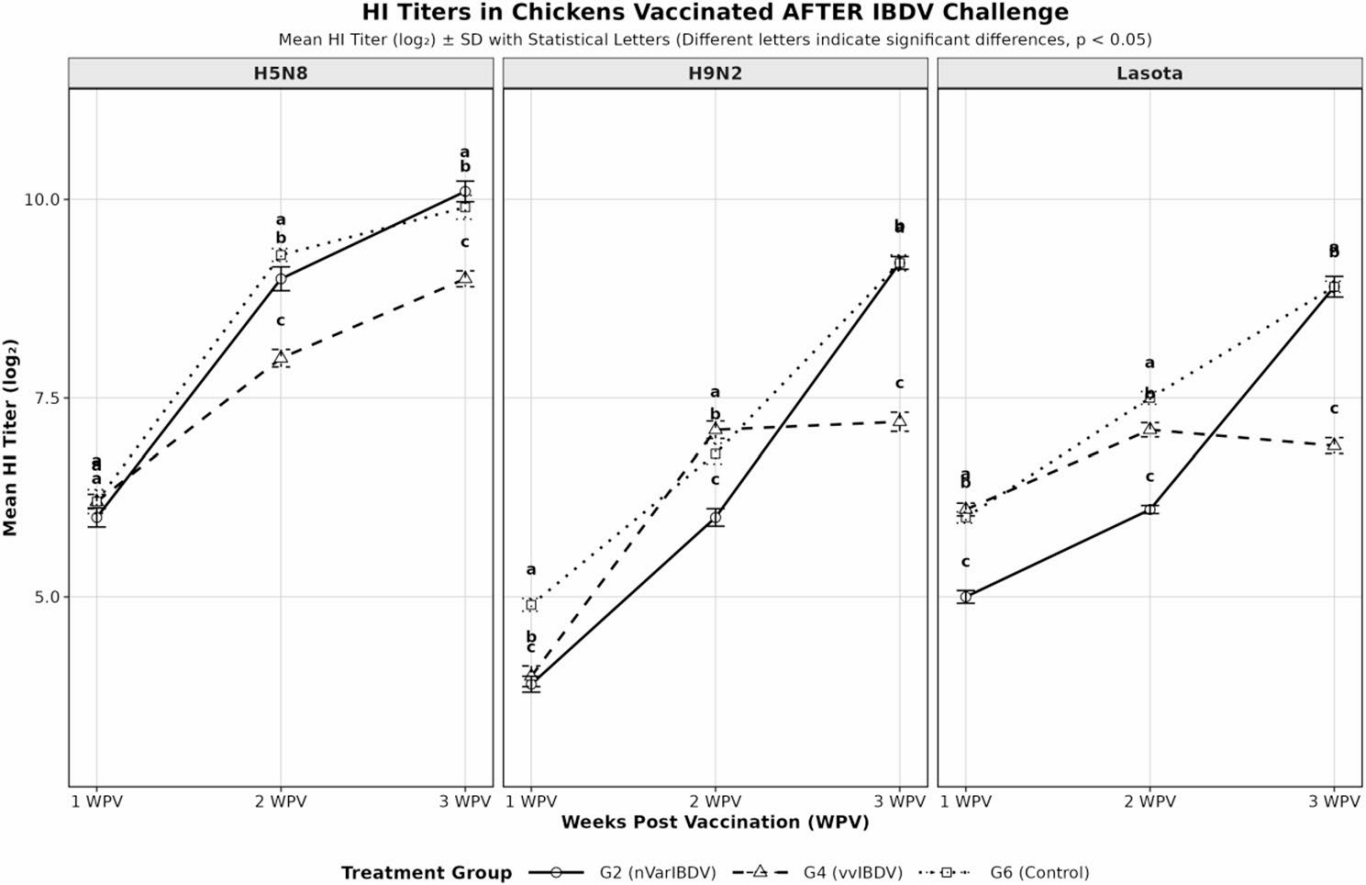
Mean HI titers (log2) for chicken groups vaccinated After challenge. Superscripts (**a**, **b**, **c**) indicate statistical differences

IBDV challenge causes significant immunosuppression in chickens (*p* < 0.05), reducing HI antibody titers by 15–40% compared to control groups. Control groups consistently maintain higher antibody levels across all time points with consistent patterns across H5N8, H9N2, and LaSota antigens as shown in figs. 8 and 9.

## Discussion

The present study investigated the pathological effects of a novel variant and very virulent IBDV strains in experimentally infected SPF chickens. The clinical signs, PM lesions, mortality rates, Bursa-to-Body Weight (B/BW) ratios, and histopathological changes were analyzed to assess the comparative virulence and immunosuppressive potential of these strains. The observed clinical signs and gross pathological findings in this study align with previous reports describing IBDV-induced immunosuppression and tissue destruction [31].

Chickens infected with the nVarIBDV strain exhibited no clinical signs (subclinical infection), but edematous and swollen bursas during early infection, followed by severe atrophy at seventh and fifteenth dpi was observed. These findings indicate that nVarIBDV strains can induce significant bursal damage, but with lower systemic severity compared to vvIBDV strains as previously described [10]. Experimental infection of the nVarIBDV strain from Japan produce subclinical disease in the specific-pathogen-free chickens during 21 dpi, but bursal atrophy was severe [25]. Recently, Infection with novel reassortant IBDV strain isolated from Iraq resulted in mild clinical disease but severe damage to lymphoid organs, causing compromised immunological responses (Fattah et al., [8]). This indicated the need for continuous monitoring by molecular techniques to detect subclinical cases of IBDV and understand virus evolution.

In contrast, the vvIBDV-infected group displayed severe systemic disease, with early onset ruffled feathers, hemorrhages in the thigh muscles, and high mortality rates (50%). The most striking gross lesion was the “sac-like” hemorrhagic bursa observed in the early phase of infection, a characteristic feature of vvIBDV infections as previously described [18]. The severe nephrosis, urate deposition, and liver congestion further confirm that vvIBDV induces multi-organ pathology beyond the bursa, contributing to systemic dysfunction and mortality [19].

The B/BW ratio and BBIX serve as a crucial indicator of IBDV-associated immunosuppression, with a lower ratio signifying severe lymphoid depletion and bursal atrophy. In the present study, the nVarIBDV and vvIBDV strains led to a significantly reduced B/BW ratio with BBIX less than 0.7. These findings are consistent with studies reporting that vvIBDV strains cause severe and irreversible bursal destruction, leading to impaired immune responses and increased susceptibility to secondary infections [12]. Another study showed that nVarIBDV infected chickens have low BBIX of 0.24 at 5 dpi indicating its drastic effect on bursa [5].

Histopathological examination revealed difference in bursal atrophy patterns between the nVarIBDV and vvIBDV strains that reflects distinct pathogenic mechanisms. The nVarIBDV strains induce progressive bursal degeneration, but they lack the acute hemorrhagic lesions observed in vvIBDV infections as previously reported [9]. This clearly reflected on the subclinical infection observed in the nVarIBDV infected group with no clinical signs reported. This aligns with previous findings that variant IBDV strains contribute to long-term immunosuppression rather than acute mortality [15, 18]. Also, these findings are consistent with previous studies that reported silent yet immunosuppressive infections caused by IBDV variant strains [9, 19].

In contrast, the vvIBDV-infected group showed early massive necrosis of bursal lymphoid follicles that progressively depleted of lymphocytes and replaced by fibrous connective tissue, indicating irreversible immunosuppression. The spleen exhibited diffuse lymphoid depletion and vasculitis, consistent with previous studies describing IBDV-induced destruction of primary and secondary lymphoid tissues [19]. Moreover, lesions observed in liver and kidneys suggesting that vvIBDV causes significant systemic inflammation beyond the bursa. Similar lesions have been documented in field cases of vvIBDV infections, where severe hepatic and renal dysfunction contribute to metabolic imbalances and increased mortality [10].

Both IBDV strains caused significant bursal atrophy, as evidenced by gross pathological changes and histopathological examination of the Bursa of Fabricius. Histopathological changes, including lymphoid depletion, fibrosis, and necrosis, have been documented in earlier reports on IBDV infections [31]. These structural alterations suggest that both viruses can severely compromise immune function, leading to increased susceptibility to secondary infections, which has been a well-established consequence of IBDV-induced immunosuppression [1]. These findings align with previous research, which reported severe bursal atrophy in infected chickens with both classical and variant IBDV strains, impacting immune development and vaccine responses in infected birds [18]. The immunosuppressive effects of IBDV, as demonstrated by bursal damage, highlight the need for effective vaccination strategies [24].

In the second experiment, we observed significant reduction in the HI titers in birds vaccinated with trivalent inactivated vaccine containing H5N8, H9N2, and Lasota antigens when challenged with either nVarIBDV and vvIBDV compared to control nonchallenged birds. Similar results observed in Chinese nVarIBDV infected chickens vaccinated with inactivated avian influenza vaccine (H5N1 and H7N9); the HI antibody titers against both H5 and H7 was significantly suppressed by nVarIBDV infection [5]. In previous study, the nVarIBDV Chinese strain caused decrease in the immune response after vaccination with live Lasota vaccine in both commercial broiler and layer SPF chickens when challenged at 21-days-old [6]. Recent study reported the significant decrease in ND antibody titers by 14 days post-challenge with Malaysian nVarIBDV strain in broiler chickens compared to the control chickens [3]. Inactivated vaccines for H9N2 and H5 avian influenza viruses (AIVs) routinely used in Egypt to control AIVs that endemically circulating in the country. Also, inactivated NDV vaccines is obligatory used in all chicken sectors in Egypt because the high environmental viral load and the high risk of NDV field challenge to decrease the expected losses. So, field exposure to nVarIBDV and vvIBDV will negatively impact the immune response not only inactivated vaccines but also live vaccines which impose high risk in increasing economic losses from NDV, AIVs and other viral outbreaks.

The extensive circulation of nVarIBDV worldwide causing subclinical disease, however it severely impacts the immune system and vaccine efficacy as highlighted in this study and several reports [3, 5, 6]. Unpublished field study showed that broilers vaccinated with H5 inactivated vaccines once or twice that exposed to nVarIBDV around 20 days-old and challenged with HPAI H5N1 virus at 28 days-old experienced 100% mortality. However, another group vaccinated once but not exposed to nVarIBDV showed 80% protection after HPAI H5N1 virus challenge. In addition to, field observations of repeated severe bacterial infection specially with E.coli and mycoplasma that not respond to any medications after detection of nVarIBDV infection by RT-PCR specially in broiler flocks. These results indicated the significant impacts of this virus poultry production with severe silent economic threat of variant IBDV strains.

For the implications for Disease Control and Prevention, the findings of this study underscore the ongoing threat posed by both nVarIBDV and vvIBDV strains in poultry production. Infection with nVarIBDV strains can induce prolonged immunosuppression, predisposing flocks to secondary infections and reduced vaccine efficacy [31]. Molecular surveillance of circulating IBDV strains is essential to track genetic mutations associated with immune escape and altered pathogenicity [18]. Moreover, Control strategies should focus on enhancing biosecurity measures, monitoring emerging IBDV variants, and improving vaccine formulations to confer cross-protection against diverse strains. Recent studies have emphasized the need for genetically engineered vaccines capable of eliciting broad-spectrum immunity against both classical and emerging IBDV strains [34]. This study highlighted the need for updating IBDV vaccines in Egypt by incorporating the circulating nVarIBDV strains in the inactivated vaccines to be used in layers and breeder flocks.

Several studies have emphasized the importance of vaccine formulation incorporating both classical and emerging variant strains to ensure broad-spectrum immunity [28]. Additional work on the development, preparation, and evaluation of an inactivated vaccine incorporating both strains, aiming to provide broad protection against nVarIBDV and vvIBDV infections is needed.

## Conclusion

The present study provides critical insights into the pathogenic effects of nVarIBDV and vvIBDV strains in experimentally infected layer SPF chickens. The comparative analysis of clinical signs, mortality rates, gross and microscopic lesions, B/BW ratios and BBIX highlights the differential impact of these two strains on poultry health and immune function. The nVarIBDV strain did not induce observable clinical signs, whereas the vvIBDV strain caused severe disease manifestations, including up to 50% mortality rates. Despite the absence of clinical signs in nVarIBDV-infected birds, its potential for immunosuppression necessitates heightened surveillance and early diagnosis to prevent undetected infections in poultry flocks specially layers and breeder flocks. Both viruses challenge shown to decrease the immune response of the inactivated vaccines either after or before vaccination. Overall, these findings emphasize the importance of continuous molecular surveillance of circulating IBDV strains to detect emerging variants. Additionally, improved vaccination strategies, incorporating both classical and variant strains, should be implemented to control the disease and decrease the economic losses in the poultry industry.

## Data Availability

The original contributions presented in the study are included in the article/supplementary material. For inquiries, please contact the corresponding author/s.

## References

[1] Abdel-Alim GA, Saif YM. Immunogenicity and antigenicity of very virulent strains of infectious bursal disease viruses. Avian Dis. 2001;45(4):924–34. 11332505

[2] Abd El-Fatah AH, Ayman D, Samir M, Eid S, Elgamal M, El-sanousi AA, Ibrahim M, AlKhazindar M, Ali MM, Amira Afify. Molecular characterization of Circulating infectious bursal disease viruses in chickens from different Egyptian governorates during 2023. Virol J. 2024;21:312. 10.1186/s12985-024-02559-9. 39616349 10.1186/s12985-024-02559-9 PMC11607947

[3] Dastjerdi PZ, Bejo MH, Rahaman NYA, Raji AA, Soontravanich R, Tai SR, Omar AR. (2025). Characterization of immunosuppression of genotype A2dB1 variant infectious bursal disease virus isolated in Malaysia using specific pathogen-free and commercial broiler chickens. Vet. World 18:799–807 Available at 10.14202/vetworld.2025.799-807 10.14202/vetworld.2025.799-807 PMC12123263 40453937

[4] Dey S, et al. Pathogenesis and molecular epidemiology of IBDV. Poult Sci. 2019;98(9):4345–53.

[5] Fan L, Wu T, Hussain A, Gao Y, Zeng X, Wang Y, Gao L, Li K, Wang Y, Liu C, Cui H, Pan Q, Zhang Y, Liu Y, He H, Wang X, Qi X. (2019). Novel variant strains of infectious bursal disease virus isolated in China. Vet. Microbiol. 230:212–220 Available at 10.1016/j.vetmic.2019.01.023 10.1016/j.vetmic.2019.01.023 30827390

[6] Fan L, Wang Y, Jiang N, Chen M, Gao L, Li K, Gao Y, Cui H, Pan Q, Liu C, Zhang Y, Wang X, Qi X. Novel variant infectious bursal disease virus suppresses Newcastle disease vaccination in broiler and layer chickens. Poult Sci. 2020;99:6542–8. 10.1016/j.psj.2020.09.037. 33248569 10.1016/j.psj.2020.09.037 PMC7704961

[7] Jackwood DJ. Advances in IBDV research. Avian Dis. 2017;61(3):219–27.

[8] Fattah ADA, Hameed SS. (2025). A novel reassortant strain of the infectious bursal disease virus (IBDV ASPVB) from Iraqi broiler farms: A first-time molecular and histopathological investigation revealing new insights. J. Adv. Vet. Anim. Res. 12:385–395 Available at 10.5455/javar.2025.l906 10.5455/javar.2025.l906 PMC12506698 41069715

[9] Jackwood DJ, Sommer-Wagner SE. Genetic characteristics of infectiousbursal disease viruses from four continents. Virology. 2010;403(1):39–50. 10.1016/j.virol.2007.03.046 17488648

[10] Jackwood DJ, Stoute ST, Crossley BM. Pathogenicity of classic and variant infectious bursal disease virus in specific pathogen-free broiler chickens. Avian Pathol. 2018;47(6):595–606.30207739

[11] Jaton J, Lozano LC, Gambini P, Ponti M, Gómez E, König GA, Zoth SC. (2024). Research Note: Characterization and phylodynamic analysis of new infectious bursal disease virus variants circulating in Argentina. Poult. Sci.:103623 Available at https://www.sciencedirect.com/science/article/pii/S0032579124002025 10.1016/j.psj.2024.103623 PMC10990894 38555757

[12] Kim IJ, et al. Immunopathogenesis of IBDV in chickens. Vet Immunol Immunopathol. 2019;210:47–55.

[13] Legnardi M, Poletto F, Talaat S, Selim K, Moawad MK, Franzo G, Tucciarone CM, Cecchinato M, Sultan H. (2023). First Detection and Molecular Characterization of Novel Variant Infectious Bursal Disease Virus (Genotype A2dB1b) in Egypt. Viruses 15 Available at 10.3390/v15122388 10.3390/v15122388 PMC10747051 38140629

[14] Liu H, et al. Host immune response to IBDV infection. Front Immunol. 2021;12:654171.

[15] Liu H, Zhang M, Han J, Qiu Y, Li L, Liu S. Genetic and antigenic evolution of emerging infectious bursal disease virus variants in China. Vet Microbiol. 2019;239:108483.31699469

[16] Lucio B, Hitchner SB. Infectious bursal disease virus: development of a standardized assay for in vitro and in vivo quantification. Avian Dis. 1979;23(1):64–74.

[17] McFerran JB, et al. Isolation and characterization of IBDV serotypes. Avian Pathol. 1980;9(3):395–404. 18770277 10.1080/03079458008418423

[18] Michel LO, Jackwood DJ. Classification of infectious bursal disease virus into genogroups. Arch Virol. 2017;162(12):3661–70. 28825213 10.1007/s00705-017-3500-4 PMC5671532

[19] Müller H, Islam MR, Raue R. Research on infectious bursal disease–The past, the present and the future. Vet Microbiol. 2012;154(1–2):1–8. 10.1016/j.vetmic.2003.08.005 14637046

[20] Reed LJ, Muench H. A simple method for estimating 50% endpoints. Am J Epidemiol. 1938;27(3):493–7.

[21] Rosenberger JK, Gelb J. Response to infectious bursal disease virus in chickens: pathogenicity and serological response. Avian Dis. 1978;22(1):263–73. 206256

[22] Salaheldin A, et al. Current epidemiology of IBDV in Egypt. Poult Sci J. 2024;63(2):230–45.

[23] Sharma JM, Dohms JE, Metz AL. Comparative pathogenesis of serotype 1 and variant serotype 1 isolates of infectious bursal disease virus and their effect on humoral and cellular immune competence of specificpathogen-free chickens. Avian Dis. 1989;33(1):112–24. 2539070

[24] Sharma JM, Kim IJ, Rautenschlein S, Yeh HY. Infectious bursal disease virus of chickens: pathogenesis and immunosuppression. Dev Comp Immunol. 2000;24(2–3):223–35. 10717289 10.1016/s0145-305x(99)00074-9

[25] Takahashi M, Oguro S, Kato A, Ito S, Tsutsumi N. (2024). Novel antigenic variant infectious bursal disease virus outbreaks in Japan from 2014 to 2023 and characterization of an isolate from chicken. Pathogens 13:1141 Available at 10.3390/pathogens13121141 10.3390/pathogens13121141 PMC11678736 39770400

[26] Thai TN, Jang I, Kim H-A, Kim H-S, Kwon Y-K, Kim H-R. (2021). Characterization of antigenic variant infectious bursal disease virus strains identified in South Korea. Avian Pathol. 50:174–181 Available at 10.1080/03079457.2020.1869698 10.1080/03079457.2020.1869698 33390030

[27] van den Berg TP. Acute infectious bursal disease in poultry. Avian Pathol. 2000;29(3):175–94. 19184804 10.1080/03079450050045431

[28] van den Berg TP, Gonze M, Meulemans G. Acute infectious bursal disease in poultry: immunosuppressive effects and vaccine prevention. Avian Pathol. 2004;33(5):409–18.

[29] von Einem J, et al. The role of VP1 in IBDV replication. J Virol. 2004;78(16):8753–61.15280483

[30] Zhang W, et al. Evolution of novel IBDV variants. Vet Res. 2022;53(1):50.35799280

[31] Eterradossi N, Saif YM. Infectious bursal disease. In: Diseases of poultry. Swayne DE, Boulianne M, Logue CM, McDougald LR, Nair V, and Suarez DL, editors. 14th ed. Wiley-Blackwell; 2020. pp. 219–46.

[32] Lukert PD, Saif YM. Infectious bursal disease. In: Diseases of poultry. Swayne DE, Boulianne M, Logue CM, McDougald LR, Nair V, Saurez DL, de Wit S, Grimes T, Johnson D, Kromm M, Prajitno TY, Rubinoff I, Zavala G, editors. 11th ed. Iowa State; 2003. pp. 161–79.

[33] OIE (World Organisation for Animal Health). Manual of diagnostic tests and vaccines for terrestrial animals. OIE; 2018.

[34] Reddy VRAP, Nazki S, Asfor A, Broadbent AJ. An infectious bursal disease virus (IBDV) reverse genetics rescue system and neutralization assay in chicken B cells. Curr Protocols. 2023;3:e639. 10.1002/cpz1.639. 10.1002/cpz1.639 PMC10108048 36622206

[35] Suvarna KS, Layton C, Bancroft JD. Bancroft’s theory and practice of histological techniques. Elsevier; 2018.

[36] Zikibayeva KB, Svanbayev AA, Akhmetsadykov NN, Kudaibergenova KN, Akhmetsadykova SN, Nurolda EN, Kydyrmanov AI. (2024). Epidemiological investigation of poultry infectious in Kazakhstan (2021–2024). Front. Vet. Sci. 11:1520606 Available at 10.3389/fvets.2024.1520606 10.3389/fvets.2024.1520606 PMC11851941 40008050

